# The Pregnancy, Arsenic, and Immune Response (PAIR) Study in rural northern Bangladesh

**DOI:** 10.1111/ppe.12949

**Published:** 2023-02-09

**Authors:** Lindsay N. Avolio, Tyler J. S. Smith, Ana Navas‐Acien, Kate Kruczynski, Nora Pisanic, Pranay R. Randad, Barbara Detrick, Rebecca C. Fry, Alexander van Geen, Walter Goessler, Ruth A. Karron, Sabra L. Klein, Elizabeth L. Ogburn, Marsha Wills‐Karp, Kelsey Alland, Kaniz Ayesha, Brian Dyer, Md. Tanvir Islam, Habibat A. Oguntade, Md. Hafizur Rahman, Hasmot Ali, Rezwanul Haque, Saijuddin Shaikh, Kerry J. Schulze, A. K. M. Muraduzzaman, A. S. M. Alamgir, Meerjady S. Flora, Keith P. West, Alain B. Labrique, Christopher D. Heaney, for the JiVitA Maternal and Child Health and Nutrition Research Project

**Affiliations:** ^1^ Department of Environmental Health and Engineering Johns Hopkins Bloomberg School of Public Health Baltimore Maryland USA; ^2^ Department of Environmental Health Sciences Columbia University Mailman School of Public Health New York New York USA; ^3^ Department of Pathology Johns Hopkins University School of Medicine Baltimore Maryland USA; ^4^ Department of Environmental Sciences and Engineering University of North Carolina at Chapel Hill Gillings School of Global Public Health Chapel Hill North Carolina USA; ^5^ Lamont‐Doherty Earth Observatory Columbia University Palisades New York USA; ^6^ Institute of Chemistry – Analytical Chemistry University of Graz Graz Austria; ^7^ Department of International Health Johns Hopkins Bloomberg School of Public Health Baltimore Maryland USA; ^8^ Department of Molecular Microbiology and Immunology Johns Hopkins Bloomberg School of Public Health Baltimore Maryland USA; ^9^ Department of Biostatistics Johns Hopkins Bloomberg School of Public Health Baltimore Maryland USA; ^10^ JiVitA Maternal and Child Health and Nutrition Research Project Gaibandha Bangladesh; ^11^ Division of Epidemiology and Community Health University of Minnesota School of Public Health Minneapolis Minnesota USA; ^12^ Institute of Epidemiology, Disease Control, and Research Dhaka Bangladesh; ^13^ Department of Epidemiology Johns Hopkins Bloomberg School of Public Health Baltimore Maryland USA

**Keywords:** arsenic, immunogenicity, influenza, micronutrients, pregnancy, prenatal exposure delayed effects, vaccines

## Abstract

**Background:**

Arsenic exposure and micronutrient deficiencies may alter immune reactivity to influenza vaccination in pregnant women, transplacental transfer of maternal antibodies to the foetus, and maternal and infant acute morbidity.

**Objectives:**

The Pregnancy, Arsenic, and Immune Response (PAIR) Study was designed to assess whether arsenic exposure and micronutrient deficiencies alter maternal and newborn immunity and acute morbidity following maternal seasonal influenza vaccination during pregnancy.

**Population:**

The PAIR Study recruited pregnant women across a large rural study area in Gaibandha District, northern Bangladesh, 2018–2019.

**Design:**

Prospective, longitudinal pregnancy and birth cohort.

**Methods:**

We conducted home visits to enrol pregnant women in the late first or early second trimester (11–17 weeks of gestational age). Women received a quadrivalent seasonal inactivated influenza vaccine at enrolment. Follow‐up included up to 13 visits between enrolment and 3 months postpartum. Arsenic was measured in drinking water and maternal urine. Micronutrient deficiencies were assessed using plasma biomarkers. Vaccine‐specific antibody titres were measured in maternal and infant serum. Weekly telephone surveillance ascertained acute morbidity symptoms in women and infants.

**Preliminary Results:**

We enrolled 784 pregnant women between October 2018 and March 2019. Of 784 women who enrolled, 736 (93.9%) delivered live births and 551 (70.3%) completed follow‐up visits to 3 months postpartum. Arsenic was detected (≥0.02 μg/L) in 99.7% of water specimens collected from participants at enrolment. The medians (interquartile ranges) of water and urinary arsenic at enrolment were 5.1 (0.5, 25.1) μg/L and 33.1 (19.6, 56.5) μg/L, respectively. Water and urinary arsenic were strongly correlated (Spearman's ⍴ = 0.72) among women with water arsenic ≥ median but weakly correlated (⍴ = 0.17) among women with water arsenic < median.

**Conclusions:**

The PAIR Study is well positioned to examine the effects of low‐moderate arsenic exposure and micronutrient deficiencies on immune outcomes in women and infants.

**Registration**: NCT03930017.

**Synopsis**

**Study question**

The Pregnancy, Arsenic, and Immune Response (PAIR) Study was designed to assess whether arsenic exposure and micronutrient deficiencies alter maternal and newborn immunity and acute morbidity following maternal seasonal influenza vaccination during pregnancy.

**What's already known**

Arsenic is associated with altered immune responses and increased risk of infection, acute morbidity, and mortality. Few studies, however, have examined arsenic and immune responses in pregnancy and infancy. Of these, few have evaluated effect measure modification by micronutrient deficiencies that influence arsenic methylation.

**What this study adds**

The PAIR Study followed a large, representative sample of mother‐infant pairs in rural northern Bangladesh. All women received the same seasonal influenza vaccine at approximately the same time during pregnancy and during the same influenza season, avoiding key confounders.

## 1 BACKGROUND

Arsenic exposure is a major threat to global health. About 140 million people worldwide are exposed to drinking water arsenic exceeding the World Health Organisation's (WHO's) guideline value of 10 μg/L.^1^ Arsenic causes bladder, lung, and skin cancers^2^ and has been associated with cardiovascular disease, diabetes mellitus, and the metabolic syndrome.^3^, ^4^ Over the past decade, multiple studies have found that arsenic was associated with altered cellular^5^ and humoral immune responses^6^, ^7^, ^8^ and increased risk of infection, acute morbidity, and related mortality.^9^, ^10^, ^11^, ^12^ Of particular concern is immunotoxicity following exposure during pregnancy and early life.^13^ However, while exposure in utero has been associated with reduced pathogen‐specific antibody responses to some childhood vaccinations^6^, ^7^, ^8^ and increased risk of respiratory and gastrointestinal morbidities in children,^14^, ^15^, ^16^, ^17^, ^18^, ^19^, ^20^, ^21^ less is known about arsenic and the immune response in pregnant women and newborns during the first months of life. In addition, arsenic methylation facilitated by one‐carbon metabolism appears to modify arsenic toxicity for certain chronic disease outcomes.^22^ Arsenic methylation typically increases during pregnancy.^23^, ^24^, ^25^ However, few studies of arsenic immunotoxicity have evaluated potential effect measure modification by micronutrient deficiencies that influence arsenic methylation in pregnant women.

The WHO recommends seasonal influenza vaccination at any stage of pregnancy to protect pregnant women and infants <6 months of age,^26^ who benefit from maternal antibodies transferred across the placenta.^27^, ^28^, ^29^ Since the risk of severe illness from infection by influenza virus is higher in pregnant women and infants,^30^, ^31^ expanding maternal vaccination against influenza is imperative. If arsenic reduces the maternal antibody response to influenza vaccine or transplacental transfer of maternal antibodies to the foetus, however, additional interventions may be needed. Yet relations among arsenic exposure, micronutrient deficiencies, antibody responses to influenza vaccination in pregnant women, transplacental transfer of maternal antibodies, and influenza‐like illness (ILI) and other acute morbidities remain poorly understood.^13^, ^32^

To better understand the influence of arsenic on immune responses in pregnant women and newborns, we established the Pregnancy, Arsenic, and Immune Response (PAIR) Study, a longitudinal pregnancy and birth cohort, in rural northern Bangladesh. The PAIR Study was designed to assess whether arsenic exposure and micronutrient deficiencies alter maternal and newborn immunity and acute morbidity following maternal seasonal influenza vaccination during pregnancy. We hypothesized that arsenic exposure and one‐carbon metabolism micronutrient deficiencies may alter maternal and newborn influenza antibody titre and avidity, measures of systemic immune responses, and respiratory and other acute morbidity outcomes among pregnant women and newborns.

## 2 METHODS

### 2.1 Study area

**FIGURE 1:**
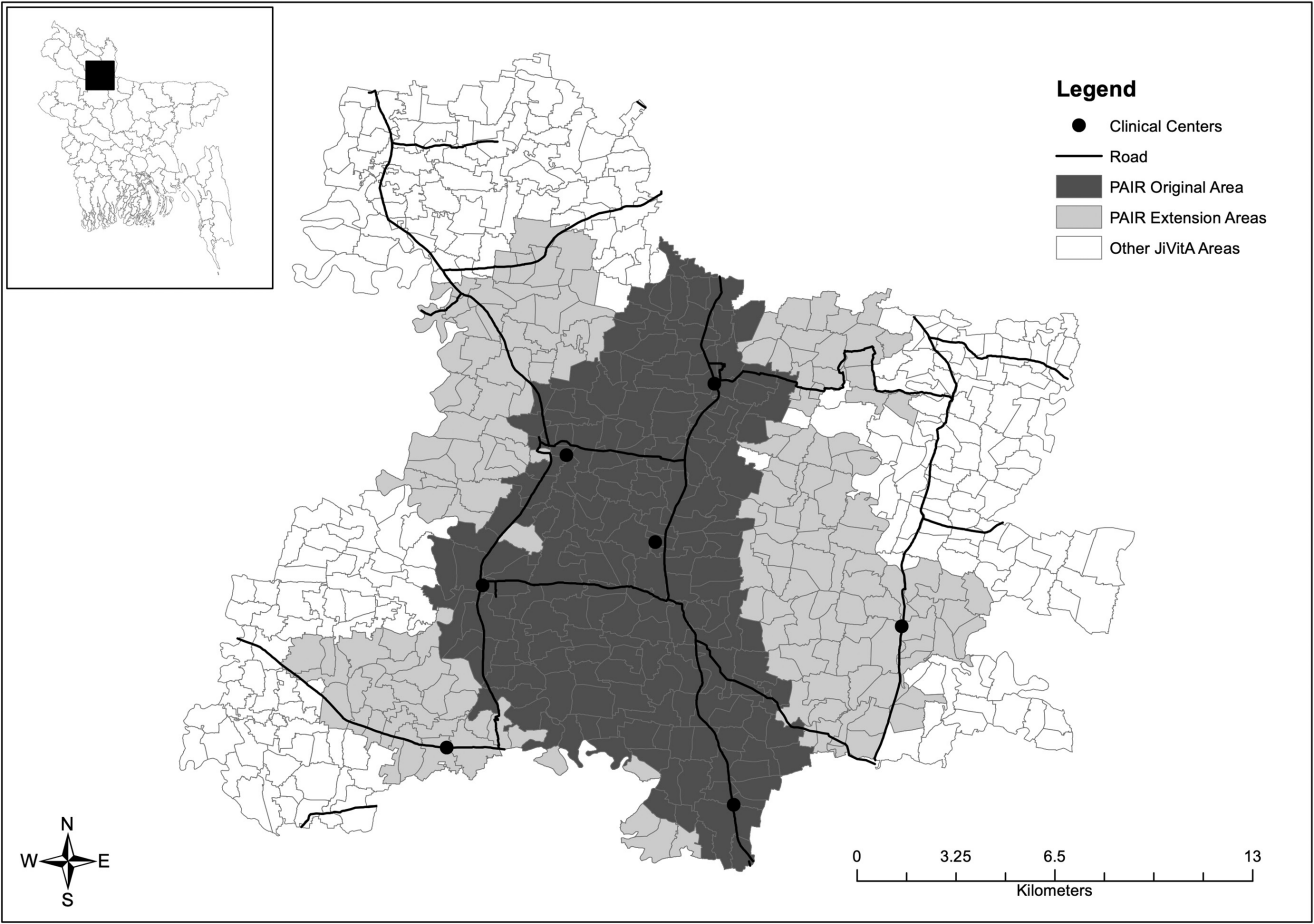
Map of the Pregnancy, Arsenic, and Immune Response (PAIR) Study area within the JiVitA Maternal and Child Health and Nutrition Research Project in northern Bangladesh, indicating the original study area (dark grey), extensions to the study area to achieve the target sample size in the same influenza season and before the Ramadan fast (light grey), and remaining JiVitA area (white). Sector boundaries are represented by light grey lines. Clinical centres where biospecimen collection was performed are noted by black circles. Major roads are indicated by black lines. The inset indicates the approximate location of the PAIR Study (black square) in Bangladesh.

The PAIR Study is based at the JiVitA Maternal and Child Health and Nutrition Research Project (JiVitA) in rural northern Bangladesh. Approximately 45 million people in Bangladesh are exposed to arsenic concentrations in drinking water >10 μg/L (the WHO guideline value) and, of these, approximately 20 million people are exposed to arsenic concentrations >50 μg/L (the Bangladesh national standard).^33^ For nearly two decades, JiVitA has been one of the largest research project sites in South Asia, covering ~650,000 people over 450 km^2^ of Bangladesh's Gaibandha and Rangpur Districts (Figure 1).^34^ JiVitA is an extensive field organisation that has conducted large trials of food and micronutrient supplements among pregnant women and children.^35^, ^36^, ^37^ A case–control study nested in a previous JiVitA micronutrient supplement trial found that urinary arsenic was associated with seroconversion to hepatitis E virus between the first trimester and 3 months postpartum among pregnant women in the study area.^9^ The PAIR Study builds on this work.

### 2.2 Enrolment and vaccination

From July 2018 to February 2019, we conducted pregnancy surveillance to identify eligible pregnant women in the study area. A married woman of reproductive age (13–45 years) was eligible for the PAIR Study if she was pregnant and 13–14 weeks of gestational age (GW) (later 13–16 GW; see below), had no pre‐existing immunodeficiency or chronic infection, had no previous or current use of immune‐altering drugs or therapies (e.g., steroids), and had not yet received an influenza vaccine for the 2018–19 influenza season.

The full JiVitA study area is divided into 566 sectors of ~150–300 households each (Figure 1). For the PAIR Study, surveillance began in 175 sectors closest to clinical centres where vaccination and biospecimen collections, including venepuncture, could be safely performed and samples could be uniformly processed (“PAIR Original Area” in Figure 1). We estimated a priori that enrolling 850 pregnant women in 13–14 GW would yield a target sample size of 400 mother‐infant pairs retained in the study to 3 months postpartum. We also determined a priori that enrolment should end by March 2019 so that pregnancies would be limited to a single influenza season and follow‐up visits at 28 days post‐vaccination would occur before the Ramadan fast (5 May to 3 June, 2019). In November 2018, seeking to achieve the target enrolment in this time window, we enlarged the study area by 163 sectors (“PAIR Extension Areas” in Figure 1) and expanded eligibility criteria to 13–16 GW.

**FIGURE 2:**
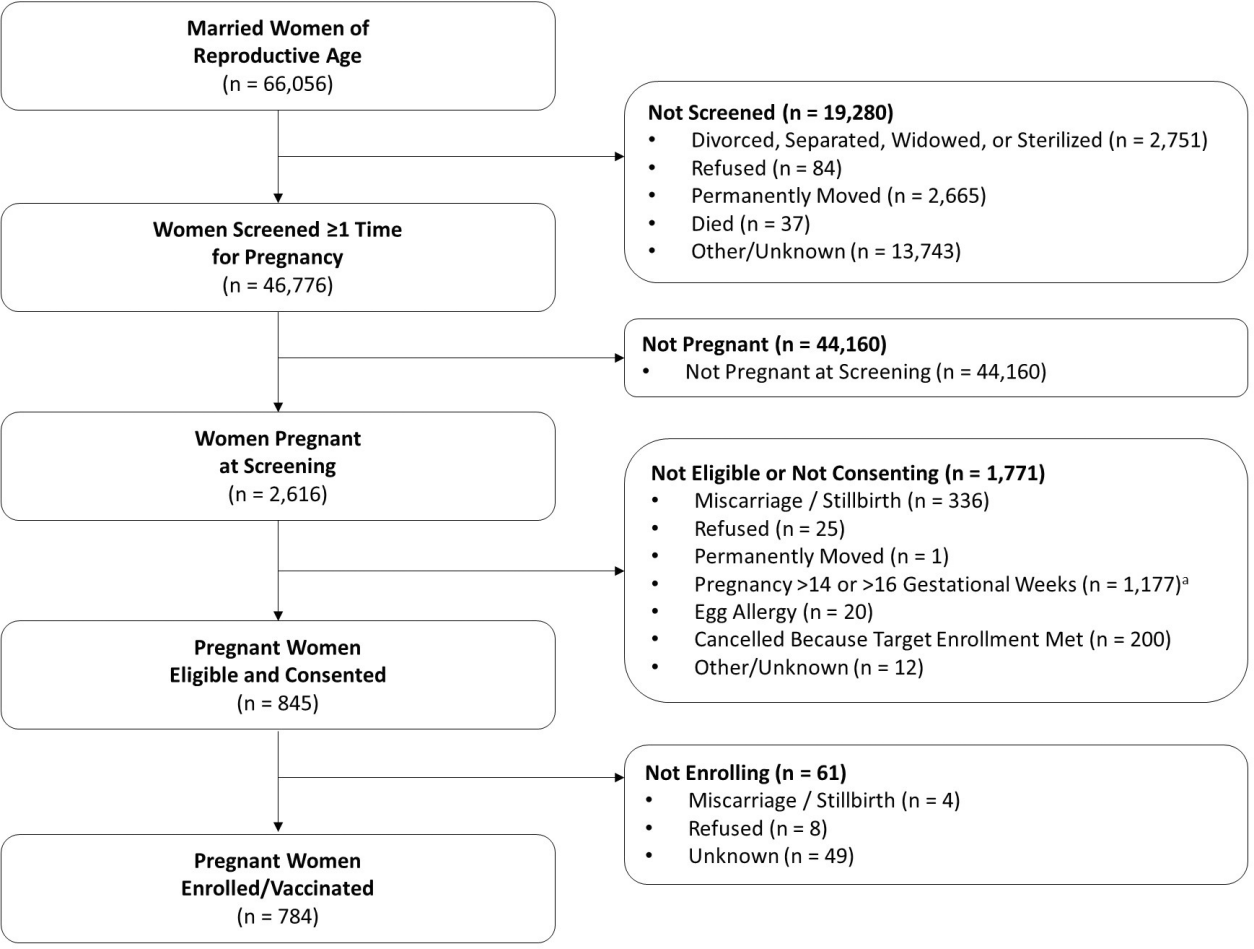
Eligibility and enrolment in the Pregnancy, Arsenic, and Immune Response (PAIR) Study, Gaibandha District, Bangladesh, 2018–2019. We conducted pregnancy surveillance from July 2018 to February 2019 and enrolled eligible pregnant women from October 2018 to March 2019. ^a^Initially, pregnant women were eligible at 13–14 weeks of gestational age (GW). In November 2018, we expanded eligibility to 13–16 GW.

A total of 66,056 married women of reproductive age living in the original or extension areas were identified by an earlier census or during pregnancy surveillance (Figure 2). During surveillance, a female community health research worker (CHRW) visited each household every 4 weeks and asked each married woman of reproductive age about the date of her last menstrual period. If the date was >30 days prior to the visit, the woman was offered a urine pregnancy test. A positive test indicated that the woman was pregnant. Gestational age was calculated from the date of her last menstrual period. We completed at least one surveillance visit to 46,776 women to identify 2616 pregnant women (Figure 2). Of these, 845 women were eligible and consented to enrolment, and 784 women enrolled in the PAIR Study between October 2018 and March 2019 (Figure 2). For logistical reasons inherent to conducting visits over a large rural area in a low‐income country, we ultimately enrolled 8 women in GW 11–12 (1.0%), 775 women in GW 13–16 (98.9%), and 1 woman in GW 17 (0.1%). At enrolment, all women received a quadrivalent seasonal inactivated influenza vaccine (VaxigripTetra, Sanofi Pasteur), which was recommended by the WHO for the 2018–19 Northern Hemisphere influenza season. The vaccine contained four inactivated influenza virus strains: A/Michigan/2015 (H1N1), A/Singapore/2016 (H3N2), B/Maryland/2016, and B/Phuket/2013.^38^ All vaccines were maintained under a cold‐chain protocol, carefully temperature‐monitored, and administered by trained nurses working under the supervision of a JiVitA physician.

Sociodemographic characteristics, including age, education, socioeconomic status, household size (number of people), and house size (number of rooms excluding the kitchen and storerooms), were similar between pregnant women who enrolled in the PAIR Study (*n* = 784) and pregnant women in the study area who did not enrol (*n* = 1519, after excluding women who were missing sociodemographic information [*n* = 313]) (Table 1). Women who enrolled tended to be younger and have higher socioeconomic status, but these differences were small (Table 1). Socioeconomic status was assessed by living standards index, which was generated by a principal components analysis of household assets and home construction materials and validated in a previous JiVitA study.^39^ The use of tube wells to obtain groundwater for drinking and cooking was nearly universal among enrolees (99.4%) and non‐enrolees (99.8%) alike (Table 1).

**TABLE 1:** Sociodemographic characteristics of pregnant women in the study area by enrolment in the Pregnancy, Arsenic, and Immune Response (PAIR) Study, Gaibandha District, Bangladesh, 2018–2019

	Enrolled (*n* = 784)	Not enrolled^a^ (*n* = 1519)
	Number (%)	Number (%)
Age (years)^b^		
<20	89 (11.4)	175 (11.5)
20–29	499 (63.6)	859 (56.6)
≥30	194 (24.7)	479 (31.5)
Missing	2 (0.3)	6 (0.4)
Maternal education		
None	92 (11.7)	212 (14.0)
Class 1–9	548 (69.9)	1065 (70.1)
Class ≥10	144 (18.4)	240 (15.8)
Missing	0 (0)	2 (0.1)
Living standards index		
<Median	360 (45.9)	792 (52.1)
≥Median	424 (54.1)	727 (47.9)
Household size (people)		
2–3	307 (39.2)	518 (34.1)
4–5	336 (42.9)	720 (47.4%)
≥6	139 (17.7)	277 (18.2)
Missing	2 (0.3)	4 (0.3)
House size (rooms)^c^		
0–1	366 (46.7)	721 (47.5)
2–3	367 (46.8)	699 (46.0)
≥4	51 (6.5)	98 (6.5)
Missing	0 (0)	1 (0.1)
Drinking water source		
Tube well	779 (99.4)	1516 (99.8)
Other	5 (0.6)	3 (0.2)

^a^ The table does not include non‐enrolled women who did not complete a separate visit to assess sociodemographic characteristics (*n* = 313).

^b^ Age as of July 2018 (start of pregnancy surveillance).

^c^ Number of rooms excluding kitchen and storerooms.

### 2.3 Study visits

We completed up to 13 in‐person visits to implement questionnaires and collect biological and environmental specimens, beginning at enrolment and continuing to 3 months postpartum, for a follow‐up period of roughly 10 months, depending on the timing and duration of each pregnancy (Tables 2 and 3). These included up to eight visits to the woman during pregnancy and up to five visits to mother‐infant pairs after live birth. At three visits (enrolment and vaccination, 28 days post‐vaccination, and 3 months postpartum), the CHRW brought participants (the mother in pregnancy, the mother‐infant pair after live birth) to a local clinical centre for detailed questionnaire and biospecimen collection. To minimise burden on mothers and infants in the neonatal period, home visits were conducted shortly after any live birth and again <1 month postpartum for further questionnaire and biospecimen collection. Of 784 women who enrolled, 744 (94.9%) completed the 28‐day post‐vaccination visit, 598 (76.3%) completed the <1 month postpartum visit, and 551 (70.3%) completed the 3‐month postpartum visit (Table 4). In total, 736 (93.9%) enrolled women had 750 live births, including 722 singletons and 28 twins. Age, gestational age at enrolment, parity, education, living standards index, household size (number of people), house size (number of rooms), height, body mass index at enrolment, and urinary arsenic at enrolment were similar among the women who enrolled and the subset of women who completed follow‐up at 3 months postpartum (Table 4).

**TABLE 2:** Visits and measurements collected from women in the Pregnancy, Arsenic, and Immune Response (PAIR) Study, Gaibandha District, Bangladesh, 2018–2020

Median week/month	Pregnancy visits (gestational weeks)								Postpartum visits (postpartum months)				Both (weekly)^b^
	14	18	22	26	30	32	34	38	<1^a^	1	2	3	
Biological specimens													
Serum	X	X							X			X	
Plasma	X	X							X			X	
Peripheral blood mononuclear cells	X	X							X			X	
Urine	X	X				X			X			X	
Oral fluid	X	X	X	X	X		X	X	X	X	X	X	
Nasal swab													X^c^
Environmental specimens													
Tube well water	X	X							X			X	
Anthropometry													
Weight	X	X				X				X		X	
Height	X											X	
Mid‐upper arm circumference	X					X				X		X	
Triceps skinfold	X											X	
Subscapular skinfold	X											X	
Vitals													
Blood pressure	X	X							X			X	
Body temperature	X	X							X			X	
Haemoglobin	X	X							X			X	
Blood typing	X												
Questionnaires													
Intentionality of pregnancy	X												
7‐ or 30‐day morbidity^b^	X	X	X	X	X	X	X	X	X	X	X	X	X
7‐day food frequency	X					X						X	
Rice and water consumption	X	X				X			X			X	
Mental health	X					X						X	
7‐day strenuous work activity	X					X						X	
Pesticide exposure history	X											X	
Poultry handling and exposure	X											X	
Handwashing and latrine use	X											X	
Tobacco use and exposure	X					X						X	
Medical history	X												
Vaccination history	X												
Household food security	X											X	
Tube well usage	X	X										X	
Altered food consumption						X							
Planned location of delivery						X							
Date and time of birth									X				
Labour and delivery									X				
Micronutrient supplements										X			
Health crisis events										X			
Resumption of menstruation												X	
Vaccination since enrolment												X	

*Note*: If a pregnancy ended before all pregnancy visits were completed, the woman did not complete the remaining pregnancy visits.

^a^ Measurements at <1 month postpartum were collected at a birth assessment visit (median day postpartum = 4) and a second visit <1 month postpartum (median day postpartum = 8).

^b^ Seven‐day morbidity was collected using weekly telephone surveillance from enrolment to 3 months postpartum and at in‐person visits. All other measurements were taken at in‐person visits only.

^c^ A nasal swab was collected if symptoms defining influenza‐like illness were reported during weekly telephone surveillance. Given limited field capacity, cases were prioritised for specimen collection based on symptom count.

**TABLE 3:** Visits and measurements collected from infants in the Pregnancy, Arsenic, and Immune Response (PAIR) Study, Gaibandha District, Bangladesh, 2019–2020

Median month	Postpartum visits (postpartum months)				Weekly^b^
	<1^a^	1	2	3	
Biological samples					
Serum	X			X	
Oral fluid		X	X	X	
Nasal swab					X^c^
Anthropometry					
Weight	X	X		X	
Length	X	X		X	
Mid‐upper arm circumference	X	X		X	
Chest circumference	X	X		X	
Head circumference	X	X		X	
Vitals					
Body temperature	X			X	
Haemoglobin				X	
Blood typing				X	
Questionnaires					
Date and time of birth	X				
Birth outcome	X				
Condition of infant at birth	X				
Newborn care practices	X				
7‐day morbidity^b^	X	X	X	X	X
Morbidities in first 28 days		X			
Breast feeding practices		X		X	
Early feeding		X			
Injury and bleeding		X			
Two‐month morbidity	X				
Child's diet	X				
Immunisation history				X	
Vitamin A supplementation				X	
Bleeding disorders				X	
Hospitalisation history				X	
Recent medication				X	

^a^ Measurements at <1 month postpartum were collected at a birth assessment visit (median day postpartum = 4) and a second visit <1 month postpartum (median day postpartum = 8).

^b^ Seven‐day morbidity was collected using weekly telephone surveillance from enrolment to 3 months postpartum and at in‐person visits. All other measurements were taken at in‐person visits only.

^c^ A nasal swab was collected if symptoms defining influenza‐like illness were reported during weekly telephone surveillance. Given the limited field capacity, cases were prioritised for specimen collection based on a symptom count.

**TABLE 4:** Characteristics [*n* (%)] of pregnant women at enrolment in the Pregnancy, Arsenic, and Immune Response (PAIR) Study, Gaibandha District, Bangladesh, 2018–2019, by completion of major study visits

Completed	Enrolment	28 days post‐vaccination	<1 month postpartum	Three months postpartum
	784 (100)	744 (94.9)	598 (76.3)	551 (70.3)
Maternal age (years)^a^				
<20	77 (9.8)	73 (9.8)	61 (10.2)	53 (9.6)
20–29	488 (62.2)	464 (62.4)	375 (62.7)	345 (62.6)
≥30	217 (27.7)	205 (27.6)	160 (26.8)	151 (27.4)
Missing	2 (0.3)	2 (0.3)	2 (0.3)	2 (0.4)
Gestational age (weeks)				
11–13	306 (39.0)	284 (38.2)	227 (38.0)	204 (37.0)
14	250 (31.9)	242 (32.5)	198 (33.1)	186 (33.8)
15	104 (13.3)	101 (13.6)	80 (13.4)	73 (13.2)
16–17	124 (15.8)	117 (15.7)	93 (15.6)	88 (16.0)
Parity				
Nulliparous	140 (17.9)	136 (18.3)	107 (17.9)	90 (16.3)
Primiparous	364 (46.4)	340 (45.7)	278 (46.5)	262 (47.6)
Multiparous	280 (35.7)	268 (36.0)	213 (35.6)	199 (36.1)
Maternal education				
None	92 (11.7)	89 (12.0)	60 (10.0)	57 (10.3)
Class 1–9	548 (69.9)	521 (70.0)	431 (72.1)	396 (71.9)
Class ≥10	144 (18.4)	134 (18.0)	107 (17.9)	98 (17.8)
Living standards index				
<Median	392 (50.0)	370 (49.7)	294 (49.2)	272 (49.4)
≥Median	392 (50.0)	374 (50.3)	304 (50.8)	279 (50.6)
Household size (people)				
2–3	307 (39.2)	289 (38.8)	224 (37.5)	207 (37.6)
4–5	336 (42.9)	321 (43.1)	259 (43.3)	238 (43.2)
≥6	139 (17.7)	132 (17.7)	113 (18.9)	104 (18.9)
Missing	2 (0.3)	2 (0.3)	2 (0.3)	2 (0.4)
House size (rooms)				
0–1	366 (46.7)	342 (46.0)	268 (44.8)	248 (45.0)
2–3	367 (46.8)	353 (47.4)	294 (49.2)	270 (49.0)
≥4	51 (6.5)	49 (6.6)	36 (6.0)	33 (6.0)
Height (cm)				
Mean (SD)	150 (5.20)	150 (5.19)	150 (5.17)	150 (5.19)
Body mass index (kg/m^2^)				
Mean (SD)	21.9 (3.44)	22.0 (3.41)	21.9 (3.36)	22.0 (3.36)
Urine arsenic (∑uAs) (μg/L)^b^				
Tertile 1 (2.98–22.5)	261 (33.3)	250 (33.6)	203 (33.9)	189 (34.3)
Tertile 2 (22.5–45.6)	261 (33.3)	248 (33.3)	193 (32.3)	175 (31.8)
Tertile 3 (46.3–451)	261 (33.3)	245 (32.9)	201 (33.6)	186 (33.8)
Missing	1 (0.1)	1 (0.1)	1 (0.2)	1 (0.2)

^a^ Age at enrolment.

^b^ Urinary arsenic is the sum of inorganic and methylated arsenic species.

In partnership with the Institute of Epidemiology, Disease Control, and Research (IEDCR), a unit of the Bangladesh Ministry of Health and Family Welfare, we conducted weekly telephone surveillance to ascertain acute morbidities in women (enrolment to 3 months postpartum) and infants (birth to 3 months postpartum). In women, we ascertained high fever with cough, high fever with a sore throat, diarrhoea, vomiting, and abdominal pain. If a woman reported a high fever with cough or sore throat, she was asked further if she had congestion, headache, or chills at the same time. In infants, we ascertained high fever with cough, diarrhoea, and vomiting. If a woman reported that her infant had a high fever with a cough, she was asked further if the infant had congestion or shortness of breath at the same time. Of 784 women enrolled in the PAIR Study and 750 infants born to them, 743 women (94.8%) and 617 infants (82.3%) participated in the surveillance.

### 2.4 Environmental specimens

We collected a water specimen from the tube well that each participant indicated was her primary source of drinking water before each centre visit and at the home visit conducted <1month postpartum (Table 2). Just prior to collection, the well was flushed for approximately 5 min. Specimens were collected into conical vials certified by the manufacturer as trace‐metal free (<1 μg/L for 20 metals, including As), and transported to the local JiVitA lab at 4–10°C. Specimens were aliquoted and stored at −20°C prior to analysis. Tube well water specimens were analysed for total arsenic (wAs) and other elements (Al, Ba, Br, Ca, Cd, Cu, Fe, K, Mg, Mn, Mo, Na, P, Pb, S, Sb, Si, Sr, U, V, W, Zn) at the Lamont‐Doherty Earth Observatory in Palisades, NY using inductively coupled plasma mass spectrometry.^40^ Empty trace metal‐free conical vials and aliquot tubes were assessed as blanks. To determine the limit of detection (LOD) for each element, we used the highest LOD across all batches. For wAs, this was 0.02 μg/L.

### 2.5 Biological specimens

Up to 22 ml of venous blood were collected from mothers at each of the three centre visits: enrolment/vaccination, 28 days post‐vaccination, and 3 months postpartum (Table 2). Up to 1 ml of capillary blood was collected from infants by heel stick during the centre visit at 3 months postpartum (Table 3). At the home visit <1 month postpartum, blood was collected from mothers and infants using the methods described for the centre visits. After blood collection, each participant received a light snack (e.g., a glucose packet). Blood specimens were transported in temperature‐controlled and ‐monitored coolers to a JiVitA laboratory. Laboratory technicians processed maternal blood to serum, plasma, and peripheral blood mononuclear cells (PBMCs), and processed infant blood to serum, on the day of collection. Serum and plasma were frozen and maintained at −80°C. PBMCs were cryopreserved following established protocols.^41^ Serum and plasma were shipped on dry ice, and PBMCs were shipped in vapour phase liquid nitrogen canisters, to the Johns Hopkins University in Baltimore, Maryland. Maternal and infant sera were tested for antibody for the influenza vaccine antigens by hemagglutination‐inhibition (HAI) assay at Sanofi Pasteur in Swiftwater, Pennsylvania.^42^ Transplacental antibody transfer will be assessed by infant‐to‐maternal HAI antibody titre ratios measured in serum collected <1 month postpartum. We also will determine avidity of IgG antibodies specific to the influenza vaccine antigens; serum cytokine and chemokine concentrations as measures of immune function; immune cell population characterisation; and T‐cell stimulation assays using the influenza vaccine antigens. We will assess plasma biomarkers relevant to one‐carbon metabolism, infection, and inflammation. Assays completed to date include plasma folate, homocysteine, vitamin B12, ferritin, and alpha(1)‐acid glycoprotein (AGP). Planned future assays include plasma albumin, vitamin D, and zinc.

Urine was collected from all mothers at the three centre visits and during the home visit <1 month postpartum, and from a subset of mothers (*n* = 468) during a home visit in late pregnancy (Table 2). Mothers were asked to provide a urine specimen in a collection cup. A CHRW immediately transferred the specimen to a container certified by the manufacturer as trace‐metal free (<1 μg/L for 20 metals, including As) and the specimens were transported to the JiVitA laboratory. Urine specimens were aliquoted and stored at −20°C prior to analysis. Urinary elements (Al, Ba, Br, Ca, Cd, Cs, Cu, Fe, K, Li, Mg, Mn, Mo, Na, P, Pb, Rb, S, Sb, Se, Si, Sr, U, V, W, Zn) were measured at the Institute of Chemistry ‐ Analytical Chemistry at the University of Graz in Graz, Austria using inductively coupled plasma tandem mass spectrometry (ICP‐MS/MS). Urinary arsenic was speciated (arsenite, arsenate, monomethylarsonic acid (MMA), dimethylarsinic acid (DMA), and arsenobetaine and other cations) by high performance liquid chromatography with ICP‐MS/MS.^43^ Empty trace‐metal free containers and aliquot tubes were assessed as blanks. The LODs for arsenic species were 0.05 μg/L. To assess arsenic exposure, we summed urinary inorganic arsenic (the sum of arsenite and arsenate; iAs), MMA, and DMA (∑uAs).^44^ To assess arsenic metabolism, we calculated proportions of urinary iAs, MMA, and DMA, relative to ∑uAs, and reported them as iAs%, MMA%, and DMA%.^22^ All urinary concentrations were corrected for specific gravity measured by refractometric determination of total solids.

We collected oral fluid, which includes saliva, oral mucosal transudate from the capillary bed, and crevicular fluid, from mothers at each centre visit, the home visit after birth, and additional monthly home visits, and from infants in all visits beginning at 1 month postpartum (Tables 2 and 3). Crevicular fluid, which flows between the gums and the teeth, is rich in antibodies and reflects the IgG profile of the serum. Oral fluid was collected by brushing the gum line with an oral‐mucosal transudate collection swab.^45^, ^46^, ^47^ Microsphere magnetic bead‐based assays will be applied to measure IgG and IgA responses specific to the influenza vaccine strains as well as a variety of endemic respiratory and gastrointestinal pathogens.^45^, ^46^, ^48^

If a mother reported influenza‐like illness (ILI) in herself or her infant during weekly surveillance, a CHRW aimed to conduct a home visit and collect a nasal swab within 24 hours of the call (Tables 2 and 3). ILI was defined as high fever with cough or sore throat in women and high fever with cough in infants.^49^ Severity was determined by calculating the sum of acute morbidity symptoms reported during surveillance. Given limited capacity, women or infants with the highest sum score were prioritised for the collection of a nasal swab. After collection by a CHRW, swabs were placed in universal transport medium intended for the preservation of viruses (Puritan UniTranz‐RT). Samples were transported to the local JiVitA lab and stored at −80°C. Nasal swabs were periodically batch‐transported to IEDCR in Dhaka, Bangladesh, in liquid nitrogen, where RNA will be extracted and tested by RT‐qPCR for influenza A and B virus, including sub‐types, according to WHO protocols to assess laboratory‐confirmed ILI.^50^

### 2.6 Anthropometry

Maternal weight (kg), height (cm), mid‐upper arm circumference (MUAC) (cm), and triceps and subscapular skinfolds (mm) were measured at enrolment/vaccination and 3 months postpartum (Table 2).^36^, ^51^, ^52^ Maternal weight was also measured at 28 days post‐vaccination, late pregnancy, and 1 month postpartum. Maternal MUAC was also measured at late pregnancy and 1 month postpartum. Infant weight, MUAC, chest circumference, head circumference, and length were recorded at the birth assessment visit (Table 3).^36^, ^51^, ^52^ Infant growth was monitored by repeating these measurements at one and 3 months postpartum.

### 2.7 Vitals

Blood pressure, haemoglobin, and body temperature of mothers were measured by a local nurse each time a mother had blood drawn (Table 2). Systolic and diastolic blood pressure were measured twice, in the left arm, with the woman in a relaxed and seated position using a WelchAllyn DuraShock DS66 Trigger Aneroid Sphygmomanometer. Haemoglobin was measured in venous blood by real‐time assay using a HemoCue Hb 301 analyser. Body temperature of infants was measured at the birth assessment and 3 months postpartum, and haemoglobin was measured at 3 months postpartum by a local nurse (Table 3). Blood type was determined using anti‐A, anti‐B, and anti‐D blood grouping reagents (Cromatest, LiNEAR). Vitals were recorded and provided to the mother after each collection. Pregnant women and infants who were severely anaemic (haemoglobin <7 g/dl)^53^ were given iron tablets and iron syrup, respectively, consistent with the study protocol and as approved by the IRB.

### 2.8 Questionnaires

Questionnaires conducted at multiple visits between enrolment/vaccination and 3 months postpartum covered a variety of topics (Table 2), including questions related to the intentionality of pregnancy, 7‐ and 30‐day morbidity including influenza‐like illness, 7‐day food frequency, mental health, 7‐day strenuous work activity, 6‐month pesticide storage and use, poultry farming and exposures, handwashing practices, tobacco use and smoke exposure, medical and vaccination history, household food security, and more. Topics related to potential sources of arsenic exposure included drinking water and rice consumption, chewing tobacco use, and husband's smoking. Additional questionnaires asked about the birth and delivery of infants, including characteristics of labour and delivery, micronutrient supplement consumption, and health crisis events. Answers to questionnaires for infants were most often provided by the mother and included morbidity, breast feeding practices, early feeding practices, infant injury and bleeding, immunisation and supplementation, and hospitalisation history and medication use (Table 3).

### 2.9 Participant incentives

All women received several participation incentives. These included the seasonal influenza vaccination, dietary counselling, antenatal care education, and a clean birthing kit (many births in the study area occur at home). As described above, we tested haemoglobin and provided severely anaemic participants with iron supplements. In women who reported severe morbidity, we provided a referral for treatment and covered the cost of transportation to receive treatment. Finally, women who completed follow‐up at 3 months postpartum received a sari.

### 2.10 Ethics approval

This study was approved by the institutional review boards of the Johns Hopkins Bloomberg School of Public Health (00008247) and the Institute of Epidemiology, Disease Control and Research (IEDCR/IRB/2017/07). All participants gave informed consent prior to enrolment.

## 3 PRELIMINARY RESULTS

**FIGURE 3:**
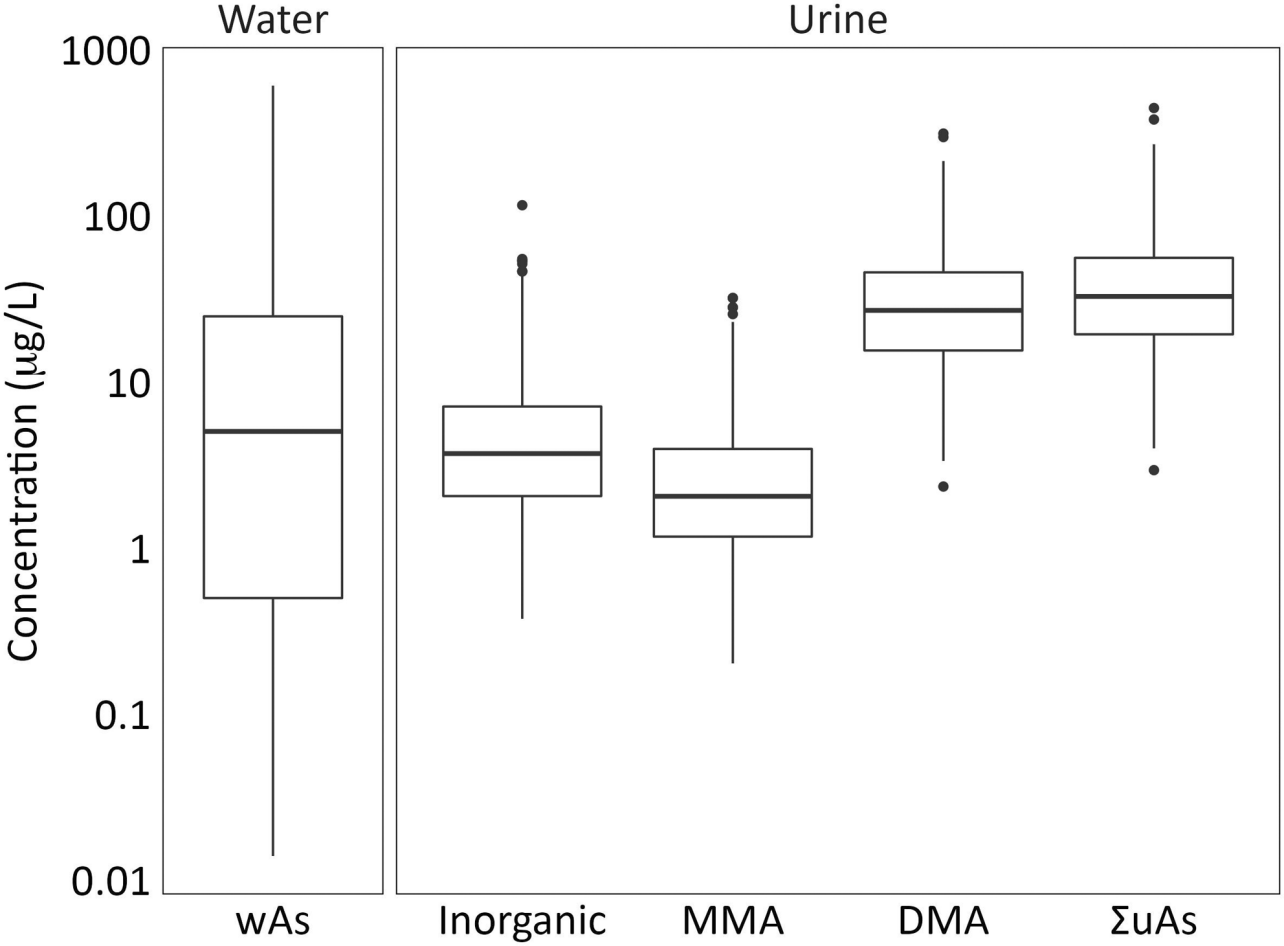
Tube well water arsenic (wAs) and urinary inorganic, monomethyl (MMA), and dimethyl (DMA) arsenic, and their sum (∑uAs), plotted on a common log scale, among pregnant women at enrolment in the Pregnancy, Arsenic, and Immune Response (PAIR) Study, Gaibandha District, Bangladesh, 2018–2019.

**FIGURE 4:**
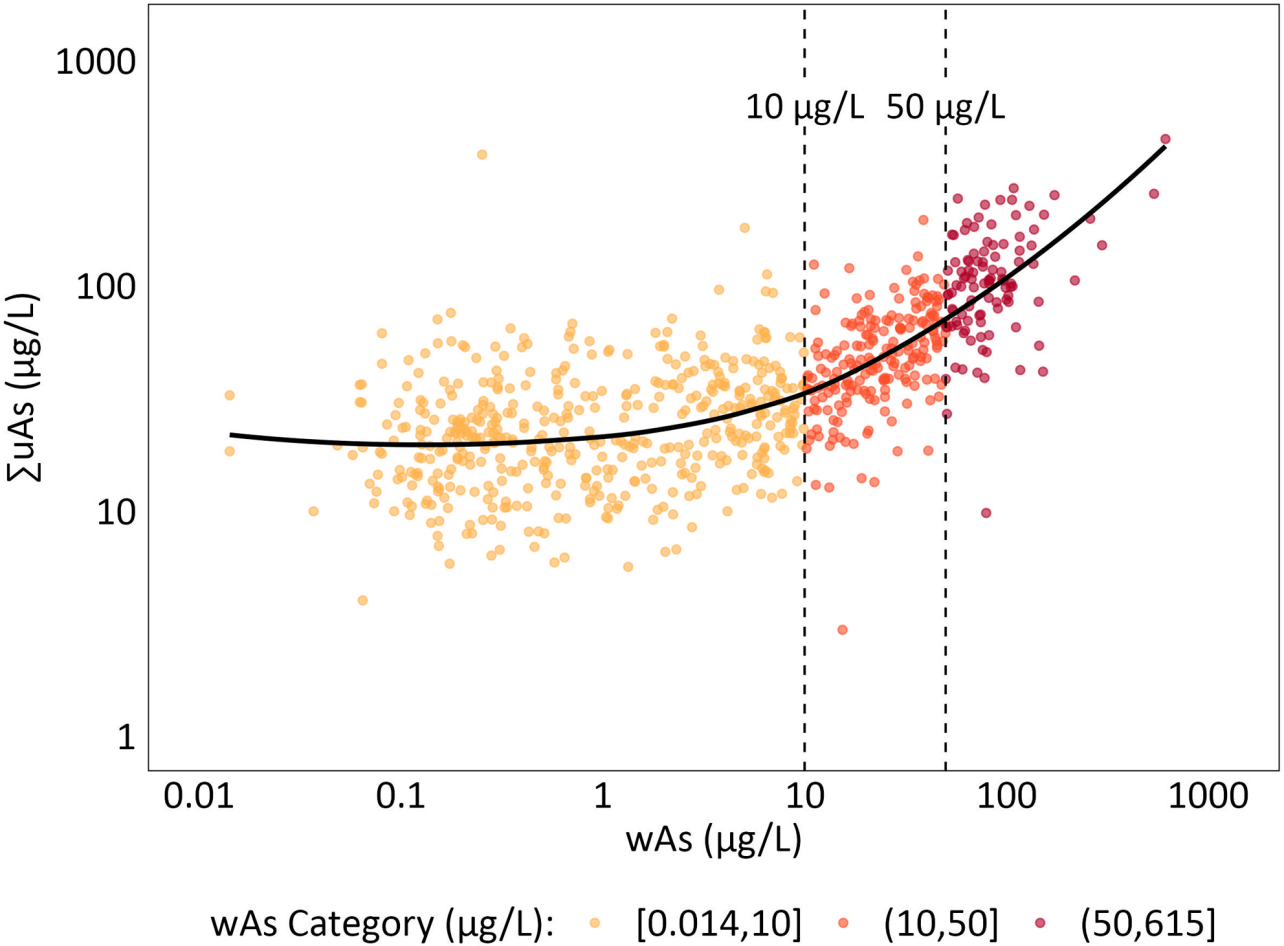
Tube well water arsenic (wAs) and the sum of urinary inorganic, monomethyl, and dimethyl arsenic (∑uAs), plotted on a common log scale, among pregnant women at enrolment in the Pregnancy, Arsenic, and Immune Response (PAIR) Study, Gaibandha District, Bangladesh, 2018–2019. A locally estimated scatterplot smoother (LOESS) is shown by the solid line. wAs standards for the World Health Organisation (10 μg/L) and Bangladesh (50 μg/L) are indicated by dashed vertical lines. wAs categories emphasise the distribution of wAs values by WHO and Bangladesh standards and indicate the sample minimum (0.014 μg/L) and maximum (615 μg/L).

Arsenic was detected (≥0.02 μg/L) in 99.7% of water specimens collected from participants' tube wells on the day of enrolment. The median (IQR) of wAs was 5.1 (0.5–25.1) μg/L (Figure 3) with 39.5% of samples above the WHO guideline value of 10 μg/L and 13.4% above the Bangladesh national standard of 50 μg/L. wAs appears to be lower in the study area than in other regions of Bangladesh where epidemiologic studies have been conducted.^54^, ^55^ Urinary iAs, MMA, and DMA were ≥LOD in all specimens collected at enrolment. The median (IQR) of their sum, ∑uAs, was 33.1 (19.6–56.5) μg/L (Figure 3). Urinary arsenobetaine (and other unretained arsenic species) levels were low (median 0.8 μg/L; IQR 0.4–1.7 μg/L), suggesting low exposure to organic arsenic from seafood in the population.^56^ Overall, ∑uAs was strongly correlated with wAs (Spearman's ρ = 0.69) (Figure 4). The correlation was slightly stronger in participants with wAs ≥ median (Spearman's ⍴ = 0.72) but weak in participants with wAs < median (Spearman's ⍴ = 0.17) (Figure 4). This suggests that, at lower concentrations of wAs, other sources of arsenic (e.g., other tube wells, rice) contribute relatively more to exposure.^57^ ∑uAs and wAs will provide complementary approaches to exposure assessment. ∑uAs integrates exposures from multiple sources, including drinking water and diet, but has a short biological half‐life and may over‐ or under‐estimate long‐term exposure.^44^ By contrast, wAs reflects a single source of exposure but is more stable over time.

## 4 COMMENT

### 4.1 Principal findings

The PAIR Study enrolled a large, representative sample of pregnant women in the late first and early second trimesters in rural northern Bangladesh and followed mother‐infant pairs to 3 months postpartum. Drinking water was the major source of arsenic exposure in the study population, as confirmed by both drinking water and urine specimens collected at enrolment. Exposure levels were low‐moderate and generally lower in our study population than in populations from other parts of Bangladesh. Urinary arsenobetaine was low, confirming that urinary arsenic reflects exposure to inorganic arsenic, not intake of organic arsenic compounds from seafood. Future work will further characterise exposure to arsenic and other metals and associations with health outcomes, including maternal antibody response to influenza vaccination, transplacental transfer of maternal antibodies, and respiratory morbidity, as well as interactions between arsenic exposure and micronutrient deficiencies in these outcomes.

### 4.2 Strengths of the study

Strengths of the PAIR Study include a large, representative sample of pregnant women and infants in rural northern Bangladesh. The women received the same seasonal influenza vaccine at approximately the same time during pregnancy and during the same influenza season. This design will enable us to assess whether arsenic exposure is associated with antibody responses to seasonal influenza vaccination in pregnant women or transplacental transfer of maternal antibodies to the foetus while avoiding potential confounders related to stage of pregnancy and annual variability in influenza viruses. Because wAs appears to be lower in the PAIR Study than in other epidemiologic studies in Bangladesh, we can assess whether low‐moderate arsenic exposure is associated with relevant maternal and infant outcomes. Biological and environmental specimens provide objective measures of arsenic exposure and immune and respiratory and other acute morbidity outcomes. Weekly telephone surveillance provides ascertainment of ILI symptoms and real‐time PCR testing of anterior nasal swabs in ILI‐symptomatic persons provides time‐resolved information on maternal and infant laboratory‐confirmed influenza A/B infection.

### 4.3 Limitations of the data

Limitations include occasional delays in study visits. While JiVitA possesses an extensive field organisation, logistical barriers to conducting frequent home visits over a large rural study area prevented some study visits from occurring as scheduled. Additionally, it was not logistically feasible to collect umbilical cord blood specimens to assess transplacental transfer of maternal antibodies. However, infant‐to‐maternal HAI antibody titre ratios measured in serum collected <1 month postpartum should provide a reasonable alternative because neonates demonstrate only a limited adaptive immune response that would allow them to generate IgG antibodies independently,^58^ and the maternal antibodies received through breast milk are IgA.

### 4.4 Collaboration

The PAIR Study was designed and conducted through collaboration among investigators at the Johns Hopkins University, Columbia University, and the University of North Carolina at Chapel Hill in the United States; the University of Graz in Austria; and the JiVitA Maternal and Child Health and Nutrition Research Project and the Institute of Epidemiology, Disease Control, and Research in Bangladesh. We welcome further collaboration. Please contact the principal investigators, Christopher D. Heaney (cheaney1@jhu.edu) and Alain B. Labrique (alabriq1@jhu.edu), for more information.

## 5 CONCLUSIONS

The PAIR Study enrolled a large, representative sample of pregnant women in the late first and early second trimester in rural northern Bangladesh. We have obtained high‐quality measurements of arsenic exposure, key micronutrients, and immune‐related outcomes in pregnant women and infants. These data will provide unique opportunities to examine effects of low‐moderate arsenic exposure and micronutrient deficiencies on immunity in pregnancy and early life. We are open to collaborations with investigators working on these issues.

## AUTHOR CONTRIBUTIONS

LNA and TJSS contributed equally to this paper. LNA: Writing – Original Draft, Data Curation, Methodology, Visualisation; TJSS: Writing – Original Draft, Data Curation, Formal Analysis, Software, Visualisation; ANA: Writing – Review & Editing, Conceptualization, Methodology, Supervision; KK: Data Curation, Resources; NP: Writing – Review & Editing, Conceptualization, Data Curation, Methodology, Project Administration; PRR: Methodology, Project Administration; B Detrick: Writing – Review & Editing, Conceptualization, Methodology; RCF: Writing – Review & Editing, Conceptualization, Methodology; AvG: Writing – Review & Editing, Investigation, Methodology; WG: Writing – Review & Editing, Investigation, Methodology; RAK: Writing – Review & Editing, Conceptualization, Methodology; SLK: Writing – Review & Editing, Conceptualization, Methodology; ELO: Methodology; MWK: Conceptualization, Methodology, Resources; K Alland: Writing – Review & Editing, Conceptualization, Methodology, Supervision; K Ayesha: Investigation, Supervision; B Dyer: Data Curation, Software; MTI: Investigation, Methodology; HAO: Investigation, Project Administration; MHR: Investigation, Methodology, Supervision; HA: Investigation, Methodology, Project Administration, Supervision; RH: Data Curation, Methodology, Supervision; SS: Conceptualization, Methodology, Project Administration, Supervision; KJS: Writing – Review & Editing, Conceptualization; AKMM: Investigation, Methodology; ASMA: Conceptualization, Methodology, Supervision; MSF: Methodology, Supervision; KPW: Conceptualization, Funding Acquisition, Supervision; ABL: Conceptualization, Funding Acquisition, Investigation, Methodology, Project Administration, Supervision; CDH: Writing – Review & Editing, Conceptualization, Data Curation, Funding Acquisition, Investigation, Methodology, Project Administration, Resources, Supervision.

## ACKNOWLEDGEMENTS

The authors thank the talented and dedicated field, laboratory, and data management teams at JiVitA and the mothers and infants who participated in the PAIR Study.

## FUNDING INFORMATION

The PAIR Study was supported by the National Institute of Environmental Health Sciences (NIEHS; R01ES026973) and by an unrestricted grant from Sanofi Pasteur (award number N/A), Lyon, France, which also provided the study vaccine and vaccine antibody testing. PAIR also benefited from common JiVitA infrastructure and staff supported by the UBS Optimus Foundation (award number N/A) and the Bill & Melinda Gates Foundation (OPP 1141435). LNA was supported by a grant from the U.S. Centers for Disease Control and Prevention, National Institute for Occupational Safety and Health to the Johns Hopkins Education and Research Center for Occupational Safety and Health (T42 OH0008428) and by a National Science Foundation Graduate Research Fellowship (DGE‐1746891). TJSS was supported by NIEHS (T32ES007141). ANA was supported by NIEHS (P42ES033719, P30ES009089).

## CONFLICT OF INTEREST

Authors report no conflicts of interest.

## Data Availability

Research data are not shared.
